# Supplementary material for: Global Evolutionary Analysis of 11 Gene Families Part of Reactive Oxygen Species (ROS) Gene Network in Four Eucalyptus Species
Source: Antioxidants (Basel). 2020 Mar 21;9(3):257. doi: 10.3390/antiox9030257 (PMC7139577; doi:10.3390/antiox9030257)
Supplement: Supplementary file 1 [file antioxidants-09-00257-s001.zip › Supplementary Figures.docx]

**
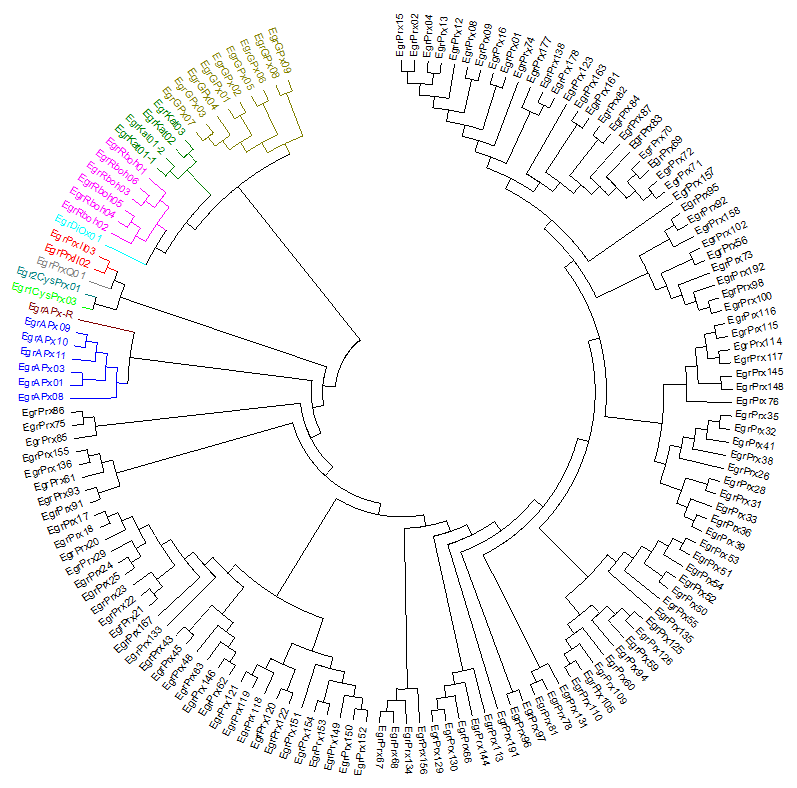
Figure S1. Phylogenetic presentation (uncompressed) of all the ROS genes in *E. grandis*.** The alignment was conducted with complete protein sequences of ROS gene using MAFFT and the phylogenetic tree was constructed using Maximum-likelihood instrument with Poisson model by Mega 6. The 11 families are indicated by different colours.


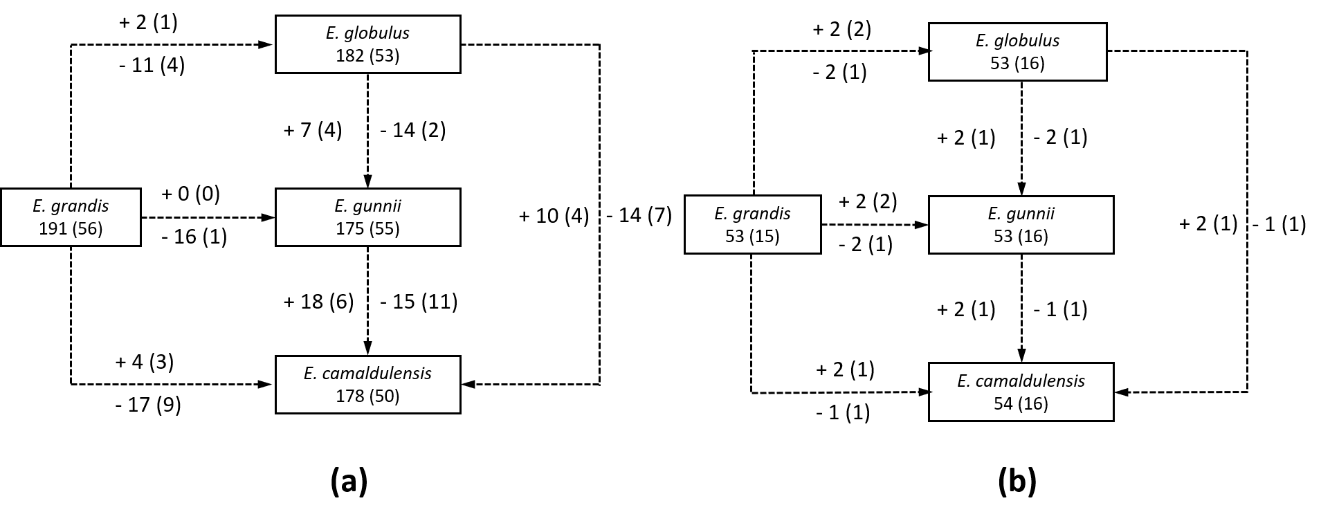


**Figure S2. Gene gain and loss during the evolutionary process.** Evolutionary changes of the number of ROS genes are shown in this figure. **(a)** Class III Prx family; **(b)** The other 10 families. The Number in rectangles together with the species name represents the isoform numbers in each organism respectively. The plus (+) and minus (–) signs indicate the numbers of genes gained and lost since speciation. The number of pseudogenes was enclosed in bracket. Take the link between *E. globulus* and *E. grandis* for an example: In *E. globulus* there are 9 Prx genes (6 pseudogenes included) containing no orthologous genes in *E. grandis* while in the mean while *E. globulus* lacks the orthologous genes of 9 Prx genes (2 pseudogenes included) in *E. grandis*.
